# 

**Published:** 1852-05

**Authors:** 


### CONTENTS

### OF NO. V.

---

### ORIGINAL COMMUNICATIONS.

### ESSAYS AND CASES.

ART. I.—*Vital Statistics of Christian County, Ky.* By W. A. EDMONDS, M. D., of Hopkinsville, Ky. - - - 369
ART. II.—An Inquiry into the Nature of the Disease called Milk-sickness. By L. P. Yandell, M. D. - - - 374
ART. III.—Midwifery Statistics. By John Swain, M. D., of Ballardsville, Ky. - - - - - - - - - 401
ART. IV.—A Clinical Lecture on Acute Pulmonary Phthisis. By DAVID W. YANDELL, M. D., one of the Consulting Physicians to the Louisville Marine Hospital. - - 403

### REVIEWS.

ART. V.—The Transactions of the American Medical Association. - - - - - - - - - - - - 409
ART. VI.—The Scientific basis of Homœopathy. By William H. Holcombe, M. D. - - - - - - - 427

### SELECTIONS FROM FOREIGN AND HOME JOURNALS.

Report of a Joint Committee of the Philadelphia County Medical Society and the Philadelphia College of Pharmacy, 429
Use of Diluted Pyroligenous Acid as a Gargle. - - - - 434
A Case of Fistula in Ano with Impacted Fæces. - - - - 535
A Case of Hernia Cerebri, - - - - - - - - 437

On the Use of the Neutral Sulphites in Diseases of the Stomach 439
The Profession of Medicine. - - - - - - - - 441
Advertising Doctors. - - - - - - - - - - 443
Arsenic Eaters. - - - - - - - - - - - 445
The Cholera of 1848 in Russia. - - - - - - - 448
Honors of Medical Men. - - - - - - - - - 449
A Case of Chorea treated by Chloroform. - - - - - 450
Chloroform in Infantile Convulsions. - - - - - - 451
On the Transformation of the two Aortæ into one in Embryonic Vertebrata. - - - - - - - - - - 451
Vital Statistics of the Medical Profession. - - - - 452
Common Salt in Ague. - - - - - - - - - 452

ORIGINAL INTELLIGENCE.

Spiritual Writings. - - - - - - - - - - 453
Medical Society of Louisville. - - - - - - - 456
Velpeau's Midwifery. - - - - - - - - - - 456
Dunglison's Medical Dictionary. - - - - - - 456
Neil and Smith's Compendium of Medicine. - - - - 457
American Medical Association. - - - - - - - 457
Graham's Chemistry. - - - - - - - - - - 458
Americian Medical Society in Paris. - - - - - - 458
Biddle's Review of Materia Medica. - - - - - - 459
Medical Students' Vademecum. - - - - - - - - 459
Meteorological Table for the month of March, 1852. - - 460

